# Improving the productive performance of growing lambs using prebiotic and probiotic as growth promoters

**DOI:** 10.1007/s11250-023-03752-8

**Published:** 2023-10-25

**Authors:** Mohsen Mahmoud Shoukry, Yasser Ahmed Abd El-Fattah El-Nomeary, Fatma Mansour Salman, Waleid Mohamed EL-Sayed Shakweer

**Affiliations:** Animal Production Department, Agricultural and Biological Research Institute, National Research Centre, 33 El-Buhouth Street, P.O: 12622, Dokki, Cairo Egypt

**Keywords:** Growth-promoters, Prebiotic, Probiotic, Productive performance, Lambs

## Abstract

The feed additives (prebiotics and probiotics) are used to stabilize the healthy gut microbiome by supporting beneficial microorganisms, thereby improving the animal growth rate. Thirty growing lambs, with around 20.50 ± 0.65 kg live weight were placed into five equal groups (6 animals each). The concentrate feed mixture (CFM) + roughage was given to the control groups. The treatments (T) of T1, T2, and T3 treatments were fed the control ration with three levels of prebiotic supplementation: 0.50, 1.00, and 1.50 g/kg CFM of mannan oligosaccharids + beta glucan, respectively. The T4 received the control ration and was supplemented with 1.0 g/kg CFM probiotic (3.0 × 10^8^ CFU/g, *Bacillus amyloliquefaciens*). The roughage was provided ad libitum, and the animals were supplemented with CFM at 2.00% of the body weight. A digestibility trial was conducted at the end of the 150-day feeding trial. The results demonstrated that increasing the prebiotic to 0.15% enhanced average daily gain and feed efficiency (*P* < 0.05) when compared to the control group. Although daily gain and feed efficiency in probiotic-fed animals were higher (*P* < 0.05) than in the control group, they were lower in prebiotic-fed lambs. The blood parameters were within normal range. The animals that received 0.10% prebiotic had the highest economic feed efficiency when compared to the other groups. Prebiotic treatment improved nutrient digestibility and nutritive values; however, the results for control and probiotic treatment were practically identical. Additionally, further research is needed to investigate the effects of prebiotics, probiotics and synbiotics as feed additives on productive and reproductive performance in ruminants.

## Introduction

Feed additives have not only been used to promote growth, but they have also been used to stabilize the beneficial gut microflora by inhibiting harmful microorganisms (Hashemi and Davoodi 2011; Abudabos et al. 2017). In the last few decades, antibiotics that are used as growth promoters in animal feed have been under severe attention since they pose a potential threat to consumers by generating antibiotic-resistant bacteria (Sultan et al. 2015). Conclusively, since 2006, the European Union has banned the supplementation of growth-promoting antibiotics in the animal diet (Khan et al. 2016). Therefore, to improve the health and productivity of animals without having any negative consequences, it is crucial to develop alternatives to antibiotic growth promoters, such as acidifiers, essential oils, probiotics, and prebiotics (Babazadeh et al. 2011). Recently, supplemented diets with phytogens as feed additives showed significant effects on growth parameters, immune response and gut health status in animals (Saeed et al. 2017).

The small intestine plays the primary role in nutrient absorption; hence, ensuring the gut's normal structure and function is effective in promoting animal health and performance (Sultan et al. 2014). Additionally, Chand et al. (2016) demonstrated that increasing the surface area of the villi increases intestinal digestion and nutrient absorption. Prebiotics are a relatively recent concept, and the premise behind prebiotics is that nondigestible dietary components like oligosaccharides are preferentially digested by bacteria known to enhance gut function. Prebiotics might encourage the growth of *lactobacilli* and *bifidobacteria* in the gut, improving the balance of the host's microorganisms. By changing the intestinal microbiota, it improves absorption, protein and fibre digestion, energy metabolism, and lowers pathogen-induced mortality (Shehata et al. 2022).

Mannan-oligosaccharide (MOS) is a commercial prebiotic product. It is mostly non-digestible carbohydrates and exists in the yeast cell wall (*Saccharomyces cerevisiae*). The cell wall of *Saccharomyces cerevisiae* contains both glucans and mannan-proteins. The MOS product, which is used in animal nutrition, was demonstrated to enhance the animal growth parameters, including feed intake and feed utilization (Nikpiran et al. 2013). The beneficial effects of MOS on the development of gut microflora were reported by Yang et al. (2008). Sadeghi et al. (2013) reported that the addition of MOS constantly elevates the caecal beneficial populations like *Bifidobacterium* and *Lactobacillus spp*, thereby decreasing the pathogenic bacteria and increasing the volatile fatty acid production (Kim et al. 2009). There is a lack of information about the use of MOS or other prebiotic types in ruminant feeding. The suggested levels for using prebiotics in ruminant feeding were not reported in the Egyptian environment (Hady et al. 2012; Ellithy et al. 2022). Therefore, the aim of the present study is to investigate the effect of different prebiotic levels on the productive performance of lamb and determine the proper level that boosts the productive performance economically.

## Materials and methods

This work was carried out at the Research and Production Station; National Research Centre (NRC) located in El-Emam Malik Village, El-Bostan, West of Nubaria; and the laboratories of the Animal Production Department, NRC, Egypt.

### Experimental treatments

The commercial products of ALTIMOS® and ENVIVA® PRO 201 BA were purchased and used for lamb feeding (Multi Vita for Animal Nutrition Company, Egypt). ALTIMOS® is a prebiotic derived from the cell walls of *Saccharomyces cerevisiae* yeast that contains 24.50% mannan oligosaccharides (MOS) and 27.7% beta-glucan (BG). ENVIVA® PRO 201 BA is a probiotic with a high concentration of *Bacillus amyloliquefaciens* (3.0 × 10^8^ CFU/g). Three levels (0.50, 1.00, and 1.50 g of prebiotic (MOS + BG)/kg CFM and one level of probiotic ENVIVA® PRO 201 BA (1.00 g PRO/kg CFM) were tested compared with control ration (0% of prebiotic or probiotic).

### Experimental animals

A total of thirty growing lambs with a live body weight of 20.50 ± 0.65 kg were divided into symmetric five groups (*n* = 6). The control group was fed a control ration based on National Research Council requirements (NRC) with no supplements (NRC 2007). Groups of T1, T2 and T3 were fed a control ration supplemented with three levels of the MOS + BG prebiotic (0.50, 1.00 and 1.50 g/kg CFM, respectively), and the T4 group was fed a control ration supplemented with 1.00 g PRO/kg CFM. The animals were fed 2.00% of their body weight from CFM and the roughage was fed ad libitum. The composition of CFM is shown in (Table 1). Table 2 shows the chemical composition of CFM and Berseem hay, while (Table 3) shows the chemical composition of various experimental rations. The lambs were fed their respective rations for 15 days as an adaptation period to adapt to the experimental rations. The feeding trial lasted for 150 days and the feed intake (FI), average daily gain (ADG) and feed conversion ratio were determined.

**Table 1:** Ingredients and composition of the concentrate feed mixture (CFM)

Ingredients	%	CP^*^	TDN^**^
Corn	50	3.9	40.0
Soybean meal	5	2.2	3.5
Cotton seed meal	20	5.0	12.0
Wheat bran	20	3.0	12.6
Premix	0.5	---	---
Limestone	3.0	---	---
Salt	1.5	---	---
Total	100	14.10	68.10

^*^CP, Crude protein; ^**^ TDN, Total digestible nutrients

**Table 2:** Chemical composition of concentrate feed mixture and Berseem hay (% on DM basis)

Item	CFM	Berseem hay
DM	88.7	90.8
OM	90.8	89.0
CP	14.6	12.9
CF	9.6	29.2
EE	3.4	1.2
NFE	63.1	45.7
Ash	9.3	11.0

*DM* Dry matter; *OM* Organic matter; *CP* Crude protein; *CF* Crude fiber; *EE* Ether extract; *NFE* Nitrogen free extract; *CFM* Concentrate feed mixture

**Table 3:** Chemical composition of different experimental rations (% on DM basis)

Item	Control	Experimental groups			PRO†
		MOS + BG^¥^			
		T1 (0.50 g/kg)	T2 (1.00 g/kg)	T3 (1.50 g/kg)	T4 (1.00 g/kg)
DM	87.8	89.0	88.9	89.0	89.0
OM	91.8	90.9	90.9	90.7	90.6
CP	15.4	15.6	15.6	15.7	15.8
CF	16.9	16.9	16.8	16.9	16.9
EE	3.2	3.4	3.2	3.9	3.4
NFE	56.4	55.0	55.3	54.1	54.5
Ash	8.2	9.1	9.2	9.4	9.4

*DM* Dry matter; *OM*, Organic matter; *CP* Crude protein; *CF*, Crude fiber; *EE*, Ether extract; *NFE*, Nitrogen free extract; *CFM*, Concentrate feed mixture; ^¥^*MOS* + *BG* prebiotic commercial product (ALTIMOS®); †*PRO* probiotic commercial product (ENVIVA® PRO 201 BA)

### Trial of digestibility

Digestibility trials were done at the end of the growth performance experiment for each group using three rams of each, where acid insoluble ash was used as a natural internal marker according to Liu (2022) to assess the nutrients' digestibility (Digestibility (%) = ((nutrient in – nutrient out) / (nutrient in)) × 100). After 3 h post-feeding, a total of 100 mL of rumen fluid was withdrawn individually by rubber stomach tube. Three layers of cheesecloth were used to filter the collected samples and the pH value was immediately measured (Orion Digital Research pH scale, Model 201). After being strained, rumen fluid samples were stored in glass bottles, then a few drops of toluene and paraffin oil were added and stored at -18°C till they were used to assess the concentrations of ammonia–nitrogen (NH_3_-N) as described by Preston (1995), and total volatile fatty acids (TVFA’s) according to Cunniff (1997). The dry matter (DM), organic matter (OM), crude protein (CP), crude fibre (CF), ether extract (EE), nitrogen free extract (NFE), and Ash were assessed in feed and feces samples in accordance with AOAC (2019) standards. Total digestible nutrients (TDN) and digestible crude protein (DCP) was calculated.

### Blood samples

At the end of the digestibility trial, blood samples were collected after 3 h post-feeding. Blood samples were centrifuged at 1350 xg for 15 min for serum separation, which was stored at -18°C until biochemical measurements were performed using a specific kit by the colorimetric method (Olympus AU 400). The blood total protein concentration was determined according to Okutucu et al. (2007), albumin according to Alcorta et al. (2018), globulin (subtracting the albumin value from the total protein value), alanine aminotransferase (ALT), aspartate aminotransferase (AST) according to Huang et al. (2006), urea according to Anton et al. (1990), and creatinine according to Jain et al. (2020).

### Economic evaluation

Based on the prices of the current study, the quick economic evaluation was achieved with the experimental supplementation that produced the highest growth performance, which was calculated as follows: sheep feed = 5.00 LE/1 kg; live body weight of lambs = 60.00 LE/1 kg; ALTIMOS (prebiotic) = 60.00 LE/1 kg; and 1 kg of ENVIVA® PRO (probiotic) = 70.00 LE/1 kg.

### Statistical analysis

Data were statistically analyzed by IBM SPSS (2011), Statistics for Windows (2011), using the following equation:

$$\mathrm{Yij}=\upmu +\mathrm{Ti}+\mathrm{eij}$$

where, Yij is the parameter under analysis ij, μ is the overall mean, Ti is the effect due to treatments on the parameter under analysis, eij is the experimental error for ij on the observation, Duncan’s multiple ranges (1955) tests were used to test the significance among means using probability level less than 0.05 (*P* < 0.05) for significance expression.

## Results

The chemical composition of the different experimental diets was very close (Table 3). Supplemented rations with MOS + BG in T2 (1.00 g/kg CFM) and T3 (1.50 g/kg CFM) groups had greater feed intake, average daily gain, growth rate and feed efficiency (inferior feed conversion ratio) compared with the control and T1 group (0.50 g/kg CFM). On the other hand, the parameters of the productive performance of lambs in T2 and T3 groups did not differ significantly. Supplemented rations with (1.00 g PRO/kg CFM, T4) recorded nearly similar values to the control group (Table 4). These results were supported by the results of nutrient digestibility trials and some ruminal and blood parameters.

**Table 4:** Effect of experimental rations supplemented with different levels of prebiotics or probiotic on feed intake, average daily gain and feed conversion of growing lambs

Item	Control	MOS + BG^¥^			PRO†	SE	*P*
		T1 (0.50 g/kg)	T2 (1.00 g/kg)	T3 (1.50 g/kg)	T4 (1.00 g/kg)		
No. of animals	6	6	6	6	6		
Feeding period, day	150	150	150	150	150		
Average initial weight (kg)	20.5	20.7	20.6	20.6	20.8	1.3	
Average final weight (kg)	47.5^c^	50.7^ab^	52.9^a^	53.0^a^	48.7^c^	1.4	< 0.05
Body wt. gain, kg/period	27.0^c^	30.0^b^	32.3^a^	32.4^a^	27.9^c^	0.8	< 0.05
Average daily gain, ADG g/h/day	180.0^c^	200.0^b^	215.0^a^	217.0^a^	186.0^bc^	3.3	< 0.05
Growth rate (%)^*^	131.7	144.9	156.6	157.3	134.1		
Total Dry matter intake, DM g/h/d	1337^c^	1385^b^	1400^a^	1415^a^	1355^bc^	70.0	< 0.05
Concentrate (g)	1003^c^	1039^ab^	1050^a^	1062^a^	1017^b^	50.0	< 0.05
Roughage (g)	334^b^	346^a^	350^a^	353^a^	338^b^	20.0	< 0.05
Feed conversion ratio (g DMI/g gain)	7.4^a^	6.9^b^	6.5^b^	6.5^b^	7.3^a^	0.57	< 0.05

a, b and c, Means in the same row with different superscripts are significantly different at (*P* < 0.05). *SE *Standard error; Significant difference at (*P* < 0.05). ^*^Total BW gain (kg)/ Initial BW (kg) × 100. ^¥^MOS + BG; prebiotic commercial product (ALTIMOS®); †*PRO *probiotic commercial product (ENVIVA® PRO 201 BA)

The experimental treatments' digestibility coefficients revealed significant differences (*P* < 0.05) between them (Table 5). The highest significant value of DM digestibility was recorded in T4 than in the control group (74.31 *vs.* 68.56%, respectively). However, there were insignificant differences between T1, T2 and T3 compared to the control group. Also, OM digestibility followed the same pattern as DM digestibility. On the other hand, the T3 group showed the highest value of CP digestibility (*P* < 0.05) than all other groups, with no significant difference with the T2 group (74.74% *vs.* 72.03%, respectively). The lowest significant value of CP digestibility was observed in the control group with 66.27%.

**Table 5:** Nutrients digestibility and nutritive values of different experimental groups

Item	Control	Experimental groups				SE	*P*
		MOS + BG^¥^			PRO†		
		T1 (0.50 g/kg)	T2 (1.00 g/kg)	T3 (1.50 g/kg)	T4 (1.00 g/kg)		
Nutrients digestibility (%)							
DM	68.6^c^	77.2^a^	77.82^a^	78.0^a^	74.3^b^	1.0	< 0.05
OM	72.0^b^	76.0^a^	77.80^a^	77.6^a^	72.6^b^	1.1	< 0.05
CP	66.3^c^	70.2^b^	72.03^ab^	74.7^a^	69.2^b^	1.5	< 0.05
CF	70.9^c^	77.4^a^	77.25^a^	78.3^a^	74.1^b^	1.4	< 0.05
EE	74.4^b^	80.3^a^	79.66^a^	80.3^a^	78.1^ab^	1.3	< 0.05
NFE	71.7^c^	78.7^ab^	79.82^a^	82.0^a^	76.9^b^	1.4	< 0.05
Nutritive values (% on DM basis)							
TDN	67.9^c^	73.4^ab^	74.37^ab^	76.4^a^	71.2^b^	1.6	< 0.05
DCP	10.2^c^	11.0^ab^	11.6^a^	11.7^a^	11.0^ab^	0.2	< 0.05

a, b and c, Means in the same row with different superscripts are significantly different at (*P* < 0.05).SE, Standard error; Significant difference at (*P* < 0.05). *DM* Dry matter; *OM* Organic matter; *CP* Crude protein; *CF* Crude fiber; *EE* Ether extract; *NFE* Nitrogen free extract; *TDN* Total digestible nutrients; *DCP* Digestible crude protein; ^¥^*MOS* + *BG* prebiotic commercial product (ALTIMOS®); †*PRO* probiotic commercial product (ENVIVA® PRO 201 BA)

The T3 group's ration supplemented with 1.50 g MOS + BG kg had a higher (*P* < 0.05) value for crude fiber digestibility (78.34%), with no significant differences between the T1 and T2 groups. On the other hand, the control group had the lowest significant value for crude fiber digestibility (70.89%) in comparison with other groups. The EE digestibility of the T1, T3, T2 and T4 groups was higher (*P* < 0.05) than that recorded for the control group. Additionally, the control group showed a significantly lower value in NFE digestibility when compared to other experimental groups. Regarding the nutritive value of TDN and DCP, the T3, T2 and T1 groups showed the highest (*P* < 0.05) nutritional values compared to the control group (Table 5).

In general, the control group recorded the lowest values for all nutrients digestibility, while T3 (1.50 g MOS + BG) showed higher values for the same nutrients digestibility, but T4 (1.00 g PRO) recorded the lowest values compared with other prebiotic tested diets (T1, T2 and T3). Therefore, nutritive values showed the same trend, where the control group recorded the lowest nutritive values compared with the other treatments, while T3 (1.50 g prebiotic) showed the highest one.

Results of some ruminal parameters of lambs fed the different experimental diets showed that lambs fed the T2 or T3 groups had a lower (*P* < 0.05) pH and a higher (*P* < 0.05) TVFA concentration than the other groups. However, ammonia N concentration was nearly similar for all treatments except for animals fed T1, which had a lower (*P* < 0.05) value compared with other groups (Table 6). Blood total protein and albumin concentrations were significantly higher in treated groups with different prebiotic levels (MOS + BG) compared to the control groups, while the rest blood parameters did not differ significantly compared to the control group (globulin, urea, creatinine, AST and ALT). However, all blood parameter values were found to be within the normal range (Table 7). In general, all ruminal and blood parameters recorded for all groups were in the normal range of the healthy animals, indicating that there is no adverse effect of the different treatments used on the health and performance of growing lambs. The economic evaluation showed that the supplementation diets (T2) with 1.0 g MOS + BG/kg CFM had given the highest net revenue based on the growth performance (Table 4).

**Table 6:** Effect of experimental rations supplemented with different levels of prebiotics or probiotic on some rumen parameters of growing lambs (3 h. after-feeding)

Item	Control	Experimental groups				SE	*P*
		MOS + BG^¥^			PRO†		
		T3 (1.5 g/kg)	T2 (1.0 g/kg)	T1 (0.5 g/kg)	T4 (1.0 g/kg)		
pH	6.7^a^	6.6^b^	6.3^c^	6.4^c^	6.8^a^	0.1	< 0.05
Ammonia-N, mg/100 mL	23.2^a^	21.2^b^	22.9^a^	22.5^a^	23.0^a^	3.1	< 0.05
Total VFA’s, meq/100 mL	13.6^b^	13.8^b^	14.3^a^	14.5^a^	13.7^b^	0.6	< 0.05

a, b and c, Means in the same row with different superscripts are significantly different at (*P* < 0.05). *SE* Standard error; Significant difference at (*P* < 0.05). ^¥^*MOS* + *BG* prebiotic commercial product (ALTIMOS®); †*PRO* probiotic commercial product (ENVIVA® PRO 201 BA)

**Table 7:** Effect of experimental rations supplemented with different levels of prebiotics or probiotic on** s**ome blood parameters of growing lambs (3 h. after-feeding)

Item	Control	Experimental groups					*P*
		MOS + BG^¥^				PRO†	
		T1 (0.50 g/kg)	T2 (1.00 g/kg)	T3 (1.50 g/kg)	T4 (1.00 g/kg)	SE	
Total protein (g/dL)	6.2^c^	6.7^a^	6.7^a^	6.8^a^	6.5^b^	0.2	< 0.05
Albumin (g/dL)	4.6^a^	4.1^b^	4.7^a^	4.6^a^	4.6^ab^	0.1	< 0.05
Globulin (g/dL)	1.6^b^	2.5^a^	2.0^ab^	2.1^ab^	2.0^b^	0.1	< 0.05
Kidney function							
Urea (mg/dL)	22.0^bc^	21.9^c^	23.3^b^	23.6^b^	24.2^a^	2.4	< 0.05
Creatinine (mg/dL)	1.5^a^	1.4^ab^	1.5^a^	1.3^b^	1.9^c^	0.5	< 0.05
Liver function							
AST (u/l)	28.9	28.3	29.5	30.2	30.7	2.7	NS
ALT (u/l)	20.7	19.1	21.1	20.9	20.6	1.2	NS

a, b and c, Means in the same row with different superscripts are significantly different at (*P* < 0.05); *SE* Standard error; Significant difference at (*P* < 0.05); *NS* non-significant; *AST* Aspartate aminotransferase; *ALT* Alanine aminotransferase; ^¥^*MOS* + *BG* prebiotic commercial product (ALTIMOS®); †*PRO *probiotic commercial product (ENVIVA® PRO 201 BA)

According to the economic evaluation's findings (Table 8), T2 supplemented with 1.0g of MOS + BG/kg is more economic efficient treatment where the ADG was increased by 35 g/day and that gave net revenue of 2.10 LE/day (0.035 × 60.00 LE for 1 kg of live body weight). The supplementation cost of the prebiotic (MOS + BG) was 0.12 LE/day, while the cost of the increased feed intake (over that of the control) was 0.24 LE/day (0.047 kg × 5.00 LE). So, the total cost of feed supplementation and the increase in feed intake was 0.36 LE/day (0.12 + 0.24 LE). Therefore, the daily net revenue = 2.10 LE – 0.36 LE = 1.74 LE/day.

**Table 8:** Economic efficiency of different levels of prebiotic (MOS + BG) and probiotic treatment

Items	Control	Experimental groups			
		MOS + BG^¥^			PRO†
		T1	T2	T3	T4
Price of prebiotic/Kg (LE/kg)	60.00				
Price of probiotic/Kg (LE/kg)	-	-	-	-	70
Feed additive (g/day)	-	0.50	1.00	1.50	1.0
Cost of feed additives (LE/day)	-	0.06	0.12	0.18	0.07
Feed intake (concentrate, Kg/day)	1.003	1.039	1.050	1.062	1.017
Price of feed intake (LE/kg)		5.00			
Increased cost of feed intake (LE/day)	-	0.18	0.24	0.30	0.07
Total cost of feed additives + the increase in feed intake	-	0.24	0.36	0.48	0.14
Price of Kg meat (LE/Kg live body weight)	60.0				
Average daily gain (ADG, g/day)	180.00	200.00	215.00	217.00	186.00
Increased ADG (g/day)	-	20.00	35.00	37.00	6.00
Income of ADG (LE/day), revenue	-	1.20	2.10	2.22	0.36
Net revenue (income, LE/day)	-	0.96	1.74	1.74	0.22

^¥^*MOS* + *BG* prebiotic commercial product (ALTIMOS®); †*PRO* probiotic commercial product (ENVIVA® PRO 201 BA)

## Discussion

Studies on prebiotics, probiotics, and synbiotics feed additives to maintain the balance of the microflora in ruminants have shown that this approach is an effective way to combat diseases that are a concern for both animals and consumers (Markowiak and Śliżewska, 2018). When compared to the control and other supplemented groups in the current study, lambs fed rations with MOS + BG (1.00 or 1.50 g/kg CFM) exhibited higher feed intake, average daily gain, feed efficiency, nutrients digestibility, and nutritional values. Additionally, the supplemented ration with probiotics (1.00 g/kg CFM) gave values that were a little bit higher than control values. Similar results have been observed in earlier studies. Growing lambs fed prebiotics or probiotics exhibited better average daily growth and ultimate weights than control groups, according to El-Mehanna et al. (2017). Moreover, Didarkhah and Dirandeh (2018) found that lambs fed rations with 2.00 g of prebiotic/head/day or 0.50 g of prebiotic/head/day had improved feed conversion compared to the control group. Recently, Zapata et al. (2021) evaluated the effects of supplemented diets with prebiotic (MOS + BG), probiotic (*Saccharomyces cerevisiae*), and the mix thereof on lambs. They reported that almost all nutrients' digestibility was increased with prebiotic and probiotic supplementation compared to the control group. Ellithy et al. (2022) also came to the conclusion that results with growing Barki lambs fed rations containing prebiotic (MOS + BG) or probiotic (*Bacillus subtilis*) were higher than those in the control group in terms of feed intake, average daily gain, feed efficiency, and nutrients digestibility. In sheep, supplemented diets with MOS improved DM, OM, and CP digestibility while decreasing ruminal ammonia concentration (*P* < 0.05). MOS can be degraded by ruminal bacteria and contributes to the stability of the ruminal microbiota (Dai et al. 2015; Wang et al. 2019). It also lengthens the intestinal villi, increasing the surface area available for nutrient absorption (Zheng et al. 2021). Lower ammonia levels in the rumen indicate increased microbial proliferation, as well as increased microbial protein synthesis and nitrogen metabolism (Diaz et al. 2018). As a result, the sheep's nitrogen utilization improved, which is consistent with the present results of nutrient utilization.

The results of the ruminal and blood parameters were within the normal range for sheep in the current investigation, demonstrating that there was no adverse effect from the different supplementations used in animal health. These results are similar to those found with Abo El-Maaty et al. (2019). Hady et al. (2012) used mannan oligosaccharides (MOS) and esterified glucomannan (EGM) as dietary supplementations (prebiotics) for Barki lambs at a level of 1 kg/ton. They concluded that both of the additives used have a positive effect on growth performance parameters with no adverse effect on rumen metabolism (pH, ammonia nitrogen and total volatile fatty acids) and blood parameters. Probiotics enhance and promote rumen metabolic development by modifying rumen functions and the microflora's fermentation activity, which enhances ruminant production efficiency (Tripathi and Karim 2011). Prebiotics inhibit dangerous pathogenic bacteria from the body while promoting the development of healthy intestinal microflora in the animal's digestive system (Semeniuk and Klebaniuk 2008). Prebiotics are non-digestible dietary components that, when taken in large enough quantities, specifically encourage the growth and/or activity of one or a select group of microorganisms in the gut (Uyeno et al. 2015). According to Dawson (1992), yeast stimulates rumen bacteria, improves lactate and ammonia consumption, and results in a moderate ruminal pH and an increase in microbial population, which in turn causes the rumen to digest fibre and produce more protein. In growing ruminants, yeast culture increased feed intake, weight gain, feed conversion (Salem et al. 2000; El-Waziry et al. 2000), and providing vitamins, yeast aids in the formation of rumen fungus (Chaucheyras-Durand and Fonty 2001). However, it has been noted that the outcomes for the use of prebiotics or probiotics as feed additives occasionally vary. This could be a result of different sources, kinds, levels, processing, usage methods, etc. These factors should be taken into consideration.

## Conclusions

From the previously obtained results, it can be concluded that the use of MOS + BG at 1.0g/kg CFM as feed additives (growth-promoters) led to an improvement in all nutrients digestibility coefficients, enhanced growth, final body weight, and feed efficiency, and improved ruminal and blood parameters, which increase the economic return of raising lambs compared to probiotics. Additionally, further research is needed to investigate the effects of prebiotics, probiotics and synbiotics as feed additives on productive and reproductive performance in ruminants.

## Data Availability

Data available by the Author by request.
